# Contract Design: Financial Options and Risk

**DOI:** 10.5334/ijic.3615

**Published:** 2018-01-12

**Authors:** Axel C. Mühlbacher, Volker E. Amelung, Christin Juhnke

**Affiliations:** 1 Health Economics and Healthcare Management, Hochschule Neubrandenburg, Neubrandenburg, DE; 2 Institute of Epidemiology, Social Medicine and Health System Research, Hannover Medical School, Hannover, DE

**Keywords:** contract design, integrated care, financial options, healthcare contracts

## Abstract

**Introduction:**

Integrated care systems as well as accountable care organisations (ACOs) in the US and similar concepts in other countries are advocated as an effective method of improving the performance of healthcare systems. These systems outline a payment and care delivery model that intends to tie provider reimbursements to predefined quality metrics. By this the total costs of care shall be reduced. When designing healthcare options contractors are faced with a variety of financial options. The costs of market utilisation are highly relevant for the conception of healthcare contracts; furthermore contract-specific investments are an obstacle to the efficient operation of ACOs.

**Methods:**

A comprehensive literature review on methods of designing contracts in Integrated Care was conducted. This article is the second in a row of three that are all published in this issue and contribute to a specific issue in designing healthcare contracts. The first dealt with the organisation of contracts and information asymmetries, while part 3 concludes with the question of risk management and evaluation. The specific research question of this second article focusses on the financial options and reimbursement schemes that are available to define healthcare contracts.

**Results:**

A healthcare contract is a relational contract, which determines the level of reimbursement, the scope of services and the quality between service providers and payers, taking account of the risks relating to population and performance. A relational contract is an agreement based upon assumption of a longer timeframe. A major obstacle to the practical implementation of healthcare contracts is the prognosis of the inflows and outflows due to the actuarial risks of the insured population. Financing conditions and reimbursement arrangements that are based on a prospectively determined fixed price, have a significant drawback: it is very difficult to take the differences in health status and the utilisation of distinct insured clientele (panel) into account.

**Discussion and Conclusion:**

The first two articles of this series on contract design have shown that complete contracts in healthcare are unrealistic. Healthcare reimbursement contracts are incomplete contracts with a high degree of uncertainty. In incomplete contracts specific contractual regulations are not made for any eventuality. For this reason it is important that the parties agree on the prevention of endogenous risks (asymmetric information after the conclusion of the contract) and on the procedure in the case of unforeseen circumstances (the risks of random, parameter risk and change risks to the healthcare program).

## Background and Objective: How to finance and reimburse new health delivery systems?

The risk structure of the providers plays a vital role in Pay for Performance (P4P). A prerequisite for optimal incentive-based service models is a (partial) dependence of the agent’s returns on the provider’s gain level.

The risk presented by the population base within a population and indicator oriented contracting must be measured and risk adjustment conducted. Extant risk-sharing modalities will be assessed and their suitability for the different regions and baseline populations.

As seen in part 1, Accountable care organisations (ACOs) and similar concepts in other countries outline a payment and care delivery model that intends to tie provider reimbursements to predefined quality metrics. By this the total costs of care shall be reduced [1].

Little is known about the contractual design and the main challenges of delegating “accountability” to these new kinds of organisations and/or contracts. Moreover, contract-specific investments are an obstacle to the efficient operation of ACOs [2].

The research questions in this series of three articles focus on how reimbursement strategies (especially Pay for Performance P4P), evaluation of measures and methods of risk adjustment can best be integrated in healthcare contracting while taking care of specific problems at the same time. For a graphic representation please see part 1 “Contract Design: The problem of information asymmetry” [3]. Part 2 of the series lays a special focus on the main components of financing and reimbursement.

A healthcare contract is a relational contract, which determines the level of reimbursement, the scope of services and the quality between service providers and payers, taking account of the risks relating to population and performance. A relational contract is an agreement based upon assumption of a longer timeframe [4,5]. Upon conclusion of the contract only a framework is agreed, the specific details are only finalized over the course of the agreed contractual period.

Healthcare contracting between providers and payers will have a major impact on the overall design of future healthcare systems. For specific problems in designing healthcare contracts especially the problem of information asymmetries as well as possible solution to this problem see “Contract Design: The problem of information asymmetry” [3].

## Financial options and risks of healthcare contracts: How to predict payments and how to reimburse services?

A major obstacle to the practical implementation of healthcare contracts is the prognosis of the inflows and outflows due to the actuarial risks of the insured population. Financing conditions and reimbursement arrangements that are based on a prospectively determined fixed price, have a significant drawback: it is very difficult to take the differences in health status and the utilisation of distinct insured clientele (panel) into account. For the parties, there are basically uncertainties regarding the time of utilisation and the amounts of actually occurring healthcare costs. If these risks are not transferred morbidity-adjusted or not sufficiently morbidity-adjusted to the care provider, there is a risk that care providers are under-or overpaid.

### Payments and Investments

#### Influencing variables on payments

If the capitation does not sufficiently match the expected individual costs there is a danger of risk selection at the system level. At this level the care provider a morbidity-specific loss probability must be assumed. The expected costs of care delivery must be estimated so that the probability of loss is limited and the actuarial risk can be replaced with an appropriate risk premium. It requires a calculation method that allows for a meaningful risk analysis and a coordinated financial management under the contract design. This can be implemented using a model of financial flows (financial model) [6].

**Figure 1:**
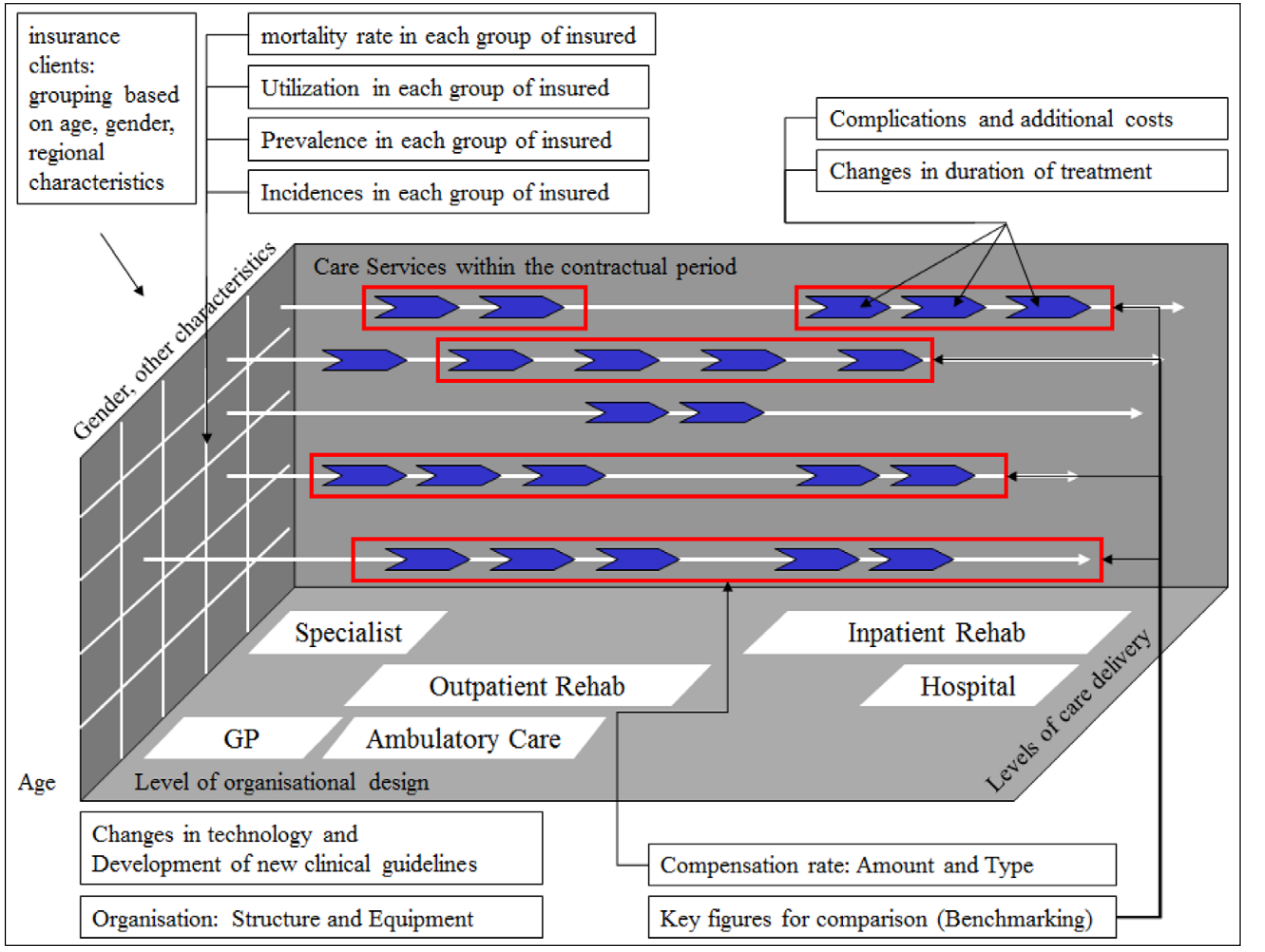
Information in the model of financial flows and risk factors (own figure).

A model of financial flows and risk factors may include the different assumptions and information, such as information about the insurance clientele: (age, gender, regional differences and other important factors for disease emergence) and assumptions on the incidence in each insured group, e. g. persons for whom a particular disease is newly diagnosed [7]. Figure 1 illustrates the assumptions and information that may be relevant to a model of financial flows and risk factors.

Such a model can assess the potential volatility in the utilisation (e.g., incidence and complications) and the variability of payments (e.g., innovation, cost inflation and demographic change in the population) and confront the proceeds. Contractual agreements regarding care contracts have an essential impact on the financial flows. Their different configuration results in different risk allocations and financial flows. The adjustment (adaptation) of contracts to the risk potential of the registered insured clientele is a crucial aspect for the potential gains or losses of both parties – payers and providers [8,9].

#### Various investment objectives

Theories of incomplete contracts deal with cases of symmetrical information with imperfect foresight and the impossibility of proving to third parties non-contractual behaviour by a party [10,11,12,13,14,15]. Before and after contractual conclusion considerable investment by integrated care organisations and ACOs have to be made in establishing healthcare networks, in the relationships to contractual partners and finally in the development of innovative healthcare reimbursement contracts. Specific investment can be made in a wide range of areas of a healthcare reimbursement contract such as:

- Investment in relationships: The service providers invest in the cooperation between them, in the certification, quality assurance and quality management, without these measures necessarily engendering higher reimbursements in terms of improved quality and profitability at the time the investment is made. The maintenance of contact with health insurers and the joint development of new forms of cooperation can also be seen as specific investments [6].
- Investment in innovative health technologies: The management of networks and the implementation of case management programs require a high level of technological support. The service providers invest in specific health technologies such as medical technology or information and communication technologies (ICT) in order to satisfy requirements for innovative healthcare chains [6].
- Investment in the development of innovative services: New forms of organisation without innovative medical care services do not meet expectations. In order to enable service providers and health insurers to incorporate successful healthcare programs into standard care, these must be developed, tested and evaluated [2].

#### Assignment of “specific” residual rights

Mixed forms between integration and economic independence can be created by the contractual assignment of residual rights to the overall yield of a transaction. Basically, it is possible to assign a contractual partner with the residual right to the overall yield of a long-term healthcare reimbursement contract, i.e. he is given the right to profit from the utilisation of the resources. In actual terms this means that the contractual partner is entitled to the amount remaining from the overall yield when all contractually agreed expenses are deducted from the health insurance premiums (after adjustment by risk structure compensation). The other contracting partners receive a reimbursement contractually agreed in advance. Assignment of residual rights can also be seen as a (partial) participation in the yield (shared saving) or risk (shared risk) of the other contracting partners. In the case of *risk sharing* too, ideal conditions cannot be assumed (see also Section Healthcare Reimbursement). Only if a contracting party is risk neutral can an ideal situation be achieved by assigning decision-making power to this party for all ex-post adjustment measures [16].

Within the context of a shared-risk capitation, neither the payers nor the service providers assume the full risk arising from a healthcare reimbursement contract. The residual rights to the profits or losses are divided between the contracting parties. This form is widespread in the USA. There is a wide range of options for risk distribution. If the contracting parties receive percentual shares in the savings of a period, this is described as *shared-savings*. A *shared-saving* or *shared-risk* agreement creates incentives for both contracting parties to control the costs and minimize contract risks. Should the costs of treating an insurance fund member exceed the reimbursement, then the provider does not lose 100% of the additional costs, but if the costs are lower than the agreed capitation, the service providers do not retain 100% of the savings either. The allocation of risks and profits can be done according to the specific investments in human or non-monetary capital of the contracting parties. This is done against the background that ideal conditions equivalent to full integration is not achievable [6].

### Reimbursement rates

Information asymmetry is not a problem if the goals of the contracting partners coincide. It is only in the case that principals and agents are pursuing different objectives that information deficits on the part of one contractual partner could lead to opportunistic behaviour. In the conception of contracts the goals of the contracting parties should be harmonized. The remuneration rules plays here a significant and in practice highly underused role. This is possible by making achievement of the service provider’s goals dependent upon achievement of the goals of the health insurer. A contract can be so conceived that upon achievement of the goals specified at the outset, the agent’s benefit is increased or so that if goals are not met there is a penalty in terms of benefit reduction. The payers are thereby enabled by way of incentive-driven contractual conception to minimise the behaviour risk of the service providers. The contracting partners should try to establish payment rules so that problems which can be foreseen ex-post are already dealt with and compensated upon contractual conclusion. The material incentives for the service providers would be optimized in this way if pursuing the goals of the health insurers/patients were in the agent’s own interests. In principle, two reimbursement methods can be distinguished [6]:

- *Directly performance-linked* reimbursement methods, in which the level of the remuneration correlates to the scope of the services performed, and
- *Indirectly performance-linked* reimbursement methods, i.e. they are not related to the number of services provided, e.g. provision of employees or per registered member.

#### Directly performance-linked remuneration methods

These can be based on the actually incurred costs, on the fee rates of the service provider and on prospectively calculated fixed rates [17]. In the first case (cost-based reimbursement) the payers assume the total costs incurred. The service providers receive part of the costs as an advance payment. At the end of the contractual term settlement is made on the basis of the service provider’s accounting compared to the services actually provided. In the case of charge-based reimbursement a fee ordinance determines which services correspond to which fees. Prospective reimbursement methods establish the reimbursement rates before performance of services commences. Compared to cost-based reimbursement, this method has no relation to the coverage of costs principle or to a fee ordinance established by the service provider. Payment is calculated in advance and negotiated before commencement of the contractual term. In general it is based upon the reimbursement of four alternative amounts [17]:

- Reimbursement can be calculated separately *per performance unit* or for each separate procedure performed on the patient (*procedure reimbursement*). Due to the high administrative costs, this form of reimbursement (particularly in the normally case of complex diagnoses and multimorbid patients) is used more often in the ambulatory sector than within the in-patient population. A doctor would behave like the “perfect agent” if he would encourage the patient to coordinate his demand for healthcare services in exactly the same way as the doctor himself would with identical information and state of knowledge [18]. The doctor in his double role – he is both service provider and decision-maker – assumes control of demand with the risk of physician-induced demand with a view to increasing his own prosperity [19].
- *Diagnosis reimbursement* remunerates the service provider according to patient diagnosis. The reimbursement is weighted according to the severity of the condition, i.e. the utilisation and cost-intensiveness of treatment. In general, case rate reimbursement is introduced on the basis of diagnosis related groups (DRGs). Here there is incentive to reduce services for a case.
- In the case of reimbursement on the basis of a *per diem rate*, service providers are reimbursed a fixed amount for each individual day of treatment, regardless of the patient’s diagnosis or severity of his condition. This form of reimbursement is nowadays only to be found in the inpatient sector (rehabilitation and psychiatric diseases). The level of the per diem rate can also be diversified, for instance with a higher reimbursement rate for time in the ICU than in other departments.
- Fixed charges for specific cases (*global pricing*) can also be agreed. Here the payers reimburse prospectively with a fixed sum for all the services provided on a particular case, regardless of whether the services are provided by one or several service providers or organisations. These specific case rates can relate to all the services provided by doctors and the hospital, for instance in the case of a cardiovascular bypass operation.

#### Indirectly performance-linked reimbursement and extent of consolidation

These parameters relate to, for instance, provision of staff members or the number of persons requiring care. *Capitation*, reimbursement on the basis of the number of insurance fund members registered (per capita rate), is paid out prospectively regardless of the scope of services. Basically, capitation guarantees a reimbursement per patient or per insurance fund member based on average utilisation of services during a certain period. The provider of a healthcare service then assumes the full risk for provision of healthcare services during a pre-defined period. Should the costs of treatment for this patient exceed the reimbursement, then the provider loses 100% of the additional costs. If the costs are under the agreed capitation, then 100% of the saving remains with the service provider.

Since reimbursement is only indirectly dependent on the actual services provided, this changes the financial framework conditions of the service providers in terms of contract risks. The reimbursement and fee settlement procedures differ in the extent to which consolidated rates are set. The extent to which consolidated rates are set for performance units is a measure of the extent to which individual procedures are summarized into reimbursement of an overall service. The level of consolidation has a massive impact on the physician’s incentive to act. It is advisable to use a combination of reimbursement components including one fixed element independent of services provided, one prospective and one performance-linked component (supply-side cost sharing) [20,21,22,23]. Generally speaking, there is a risk with incentive contracts that the performance incentives set do not fully correspond to the goals of the principal. Activities which are not contained within the incentive structure, i.e., have no direct influence on the agent’s benefit, will be neglected. The health insurers as custodians of the insurance fund members must consider both the quality and the economic feasibility of treatment as target criteria. Different forms of reimbursement create different incentives [9].

#### Reimbursement rates

Regardless of the payer there is only a limited number of ways in which care providers can be reimbursed for their services. As seen, the alternative reimbursement approaches can roughly be divided into reimbursement approaches with direct and indirect performance link [24].

In addition to a salaried employee and the payment by a fixed salary, there is a wide range of forms of reimbursement. Reimbursement forms can be classified according to the following distinguishing features (see Table 1):

- Relation to performance (performance-linked)
- Calculation Time
- Time of payment (prospective vs. retrospective)
- Level of generalization and scope
- Extent of services (payment of networks or individual providers)

**Table 1:** Overview on reimbursement methods (own table).

Reimbursement forms	Performance-linked	Time of calculation	Time of payment	Level of generalization	Profundity/extent of services

Salary	Indirect	Calculation is independent from actually provided services	Payment is independent from actually provided services	Encompassing all services of the employee	Care provider
Fee for service	Direct	Prospective	Retrospective; cost reimbursement principle or fee schedule	Individual service	Care provider
Case rate	Direct	Prospective	Retrospective	Case, organisation-internally	Care provider
Complex case rate	Direct	Prospective	Retrospective	Case, across organisations	Network of care providers
Contact capitation	Indirect	Prospective	Retrospective	Full services, indication-specific	Network of care providers
Capitation	Indirect	Prospective	Prospective	Full service provision	Network of care providers

The diversity and potential of forms of reimbursement systems are large. In “fee for service” costs spend on a specific treatment case may be determined by the insurance (payer) only after the care delivery (retrospective). Whereas in the remuneration by case rates a price for a particular complex of services (treatment case or full coverage) is calculated as a fixed amount before the actual care delivery takes place (prospectively). Diagnostic data of a base year are used to forecast the cost of the following year. With retrospective data – compared to the prospective calculation – the differences in the individual claims are better modelled as unforeseen expenses can be included in the calculation. Payment for the care providers in case rates (fee per case, complex case rate, capitation, Contact capitation) is independent from the care provision, i.e., regardless of which services the patient utilises within the contractually agreed scope of services during the contract period. Depending on the level of generalization the extent of services describes how individual providers or networks of providers will be reimbursed. For a systematic overview on existing reimbursement methods in healthcare please see [25,26,27]:

#### Fee for service versus case rates

Because of the largely free design reimbursement systems are often designed in a way that the care providers are motivated to make the care effectively and avoid volume increases [28]. The fee- for-service features no positive effect, because a specific, well-defined medical service is paid at a fixed fee in this form of remuneration, with duration and difficulty being additionally considered. Hence, medically not-indicated services and the incentive to expanse care are promoted [29].

The interest of health insurance is therefore more in releasing the budget responsibility and in arranging lump payment. The economic incentive here is in of the minimization volume [30], since all packages are calculated independently of the actual costs [31]. In such a system, the reimbursement is based on the number of patients who have called/visited an office in an agreed time period (quarter or year). As a result, doctors will try to treat each case as cost-saving as possible to take a large share of the package without proper consideration in work [30]. Case rates induce healthcare providers to deal intensively with the management of services. Their thinking is process-, customer-and quality-oriented and motivates to network [32]. In addition, a lump-sum compensation increases transparency, cost awareness and bridges sectorial boundaries [33]. However, there is also a risk that providers offer inferior or inadequate services in order to achieve savings. Even the development of risk selection is feasible. As a result mostly young and healthy insured would be asked to participate in integrated care, while insured with poor risks remain in the standard care [34,35].

## Discussion and Conclusion

Competition between the various organisations of the service (managed competition) can be expressed in two forms: as competition for health insurance members or as competition for healthcare reimbursement contracts [36].

In order to achieve the best possible level of efficiency, the exogenous and endogenous contract risks must be taken into account during conception of the contractual relations between health insurers and organisations of service providers. If healthcare reimbursement contracts are conceived so as to take adequate account of incentives and risks the economic effects of selective contracting and increased welfare might be the result.

The first two articles of this series on contract design have shown that complete contracts in healthcare are unrealistic. Healthcare reimbursement contracts are incomplete contracts with a high degree of uncertainty. In incomplete contracts specific contractual regulations are not made for any eventuality. For this reason it is important that the parties agree on the prevention of endogenous risks (asymmetric information after the conclusion of the contract) and on the procedure in the case of unforeseen circumstances (the risks of random, parameter risk and change risks to the healthcare program). Since in some cases the behaviour of the parties cannot be verified by third parties, one can benefit from the specific investments of others (hold-up). Opportunistic action is to be expected if one of the parties has an information advantage. Strategic behaviour on the basis of moral hazard and hold-up can be described as endogenous risks. Uncertainties are based on the risk of random, and changing parameters can be seen as an endogenous risk since neither party can control these risks completely. It is also not possible to integrate these risks in the contract or to eliminate these risks by the parties. To control these risks, healthcare providers/payers should provide reimbursement contracts that include structured models for renegotiation and risk sharing/shared-savings.
